# Clinical and genomic profiling of a patient with a *de novo* ring chromosome 18: a case report highlighting autoimmune and neurological implications

**DOI:** 10.1186/s13039-024-00700-5

**Published:** 2024-12-05

**Authors:** Annalaura Montanari, Paola Caforio, Annalisa Paparella, Paola Casieri, Maria Cristina Nuzzi, Maria Fatima Antonucci, Claudia Rita Catacchio, Marilina Tampoia, Mattia Gentile, Roberta Bucci, Valerio Cecinati, Angelo Cellamare, Francesca Antonacci

**Affiliations:** 1 Dipartimento di Bioscienze, Biotecnologie e Ambiente, Università degli Studi di Bari “Aldo Moro”, Bari, Italy; 2 U.O.C Patologia Clinica - Sezione di Genetica Medica, Ospedale SS. Annunziata Taranto - ASL Taranto, Taranto, Italy; 3 U.O.C. Laboratorio di Genetica Medica, PO Di Venere - ASL Bari, Bari, Italy

## Abstract

Ring chromosome 18 (r(18)) is a rare chromosomal abnormality characterized by the circular rearrangement of chromosome 18, which presents significant challenges in genotype-phenotype correlations due to variability in deletions across the 18p and 18q arms. We report the case of a pediatric patient with a *de novo* ring chromosome 18, diagnosed by karyotype analysis and confirmed by high-resolution SNP arrays. The patient exhibited pathogenic copy number variants (CNVs) in the 18p11.32p11.22 and 18q23 regions, involving 36 and 10 OMIM genes, respectively. Clinically, the patient presented with hypothyroidism secondary to autoimmune thyroiditis, autoimmune hepatitis type II, and genetic predisposition to celiac disease and insulin-dependent diabetes mellitus (IDDM) along with notable dysmorphic features. The 18q microdeletion encompasses the *MBP* gene, involved in the development and functionality of the nervous system, as supported by hypotonia and gliosis shown by the MRI. This case highlights the complex interplay between genetic imbalances on chromosome 18 and autoimmune phenotypes, emphasizing the need for ongoing research to elucidate underlying mechanisms and optimize clinical management for individuals with r(18).

**Supplementary Information:**

The online version contains supplementary material available at 10.1186/s13039-024-00700-5.

## Introduction

Chromosomal abnormalities can significantly disrupt gene balance, leading to various genetic disorders. Among these, ring chromosomes are rare anomalies that typically result when both arms of a chromosome break and then fuse to form a ring structure [1–3]. This often leads to the loss of the terminal regions of both the short and long arms of the chromosome, resulting in a hemizygous condition for genes located within the deleted regions [4–6]. Although such events can occur on any chromosome, chromosome 18 is particularly susceptible to forming ring structures, known as ring chromosome 18 (r(18)) [3]. This structure typically arises as a result of *de novo* errors during early embryonic development or maternal gametogenesis [2, 7], with rare cases of familial transmission reported [4, 8].

Individuals with r(18) typically present with features from both 18p and 18q deletion syndromes, which can include craniofacial dysmorphisms, developmental delays, and a range of systemic anomalies such as cardiac, skeletal, and immunological disorders [2, 4–6, 9, 10]. The broad phenotypic variability observed in r(18) patients primarily arises from differences in the size of the chromosomal deletions. Recent advances in genetic testing, including single-nucleotide polymorphism (SNP) arrays, have improved the precision of breakpoint mapping, which aids in more accurate genotype-phenotype correlations and enhances clinical diagnosis and patient management [3, 11]. In this study, we describe the case of a pediatric patient with a *de novo* r(18), including a comprehensive clinical and genetic characterization. Our analysis particularly focuses on the autoimmune manifestations associated with this chromosomal anomaly, exploring the potential role of genes on chromosome 18 in autoimmune processes.

## Case report

The proband is the second child born to healthy, non-consanguineous parents at 39 weeks of gestation via spontaneous delivery following an uneventful pregnancy. The family history is significant for diabetes and hyperthyroidism.

At birth, the patient’s auxological parameters were normal but at 9 months difficulties with chewing and swallowing and poor height-weight growth occurred.

The patient was referred at the age of 2 years and 7 months due to global developmental delay and hypertransaminasemia, first identified at the age of 23 months.

Early signs of psychomotor developmental delay were evident by 16 months, together with microcephaly, global developmental delay, hypotonia with ligamentous hyperlaxity, motor instability, and an eating behavior disorder.

During clinical evaluation, several dysmorphic features were identified in the proband, including an asymmetric face, flat profile, sparse eyebrows, long eyelashes, periorbital fullness, epicanthus, hypertelorism, large ears, a depressed nasal bridge, a broad nasal tip, an asymmetric mouth and dental arches, thin upper lip, high palate, protruded tongue, micrognathia, stumpy neck, umbilical hernia, small hands and feet, bilateral flat foot, clinodactyly of the IV toe and short stature.

Abdominal ultrasound, ophthalmological and ORL examinations were normal. Magnetic Resonance Imaging (MRI) of the brain revealed the presence of some millimetric hyperdense areas in FLAIR/T2, iso-hypointensein T1 and without impregnation after intravenous contrast medium administration in the deep white matter of the corona radiates and semioval centers. These findings were interpreted as indicative of gliotic reparative processes.

Extensive laboratory workup revealed positive AbTPO and AbTG, along with persistent hypertransaminasemia.

An endocrinological examination and thyroid ultrasound confirmed the diagnosis of hypothyroidism secondary to autoimmune thyroiditis.

HLA DRB1/DQB1 screening for celiac disease indicated the presence of the DR7.DQ2 haplotype but the absence of homozygous DQ2 status, suggesting a genetic susceptibility to celiac disease.

During hospitalization for hypertransaminasemia, the patient underwent extensive hematological and autoantibody testing, revealing elevated TSH (8.92 mUI/L), high titers of AbTG (110.8 IU/ml) and AbTPO (> 13000.0 IU/ml), and positive LKM1 (liver-kidney microsomal type 1) antibodies. This serological profile is indicative of autoimmune hepatitis type II.

A liver biopsy further confirmed the diagnosis of autoimmune hepatitis type II, revealing typical histopathological findings: portal and periportal inflammation, cholangitis, and fibrotic expansion.

At 3 years and 2.6 months, the patient exhibited auxological parameters lower than the 3rd centile with a stable growth pattern, selective eating behavior and persistent developmental delays.

She is currently undergoing periodic multidisciplinary (neuropsychiatric, pediatric gastroenterological, hepatological and endocrinological) follow-ups to monitor her condition and receiving regular treatment with levothyroxine, corticosteroids, azathioprine and ursodeoxycholic acid and speech and psychomotor therapy.

## Results

### Karyotype analysis

**Fig. 1:**
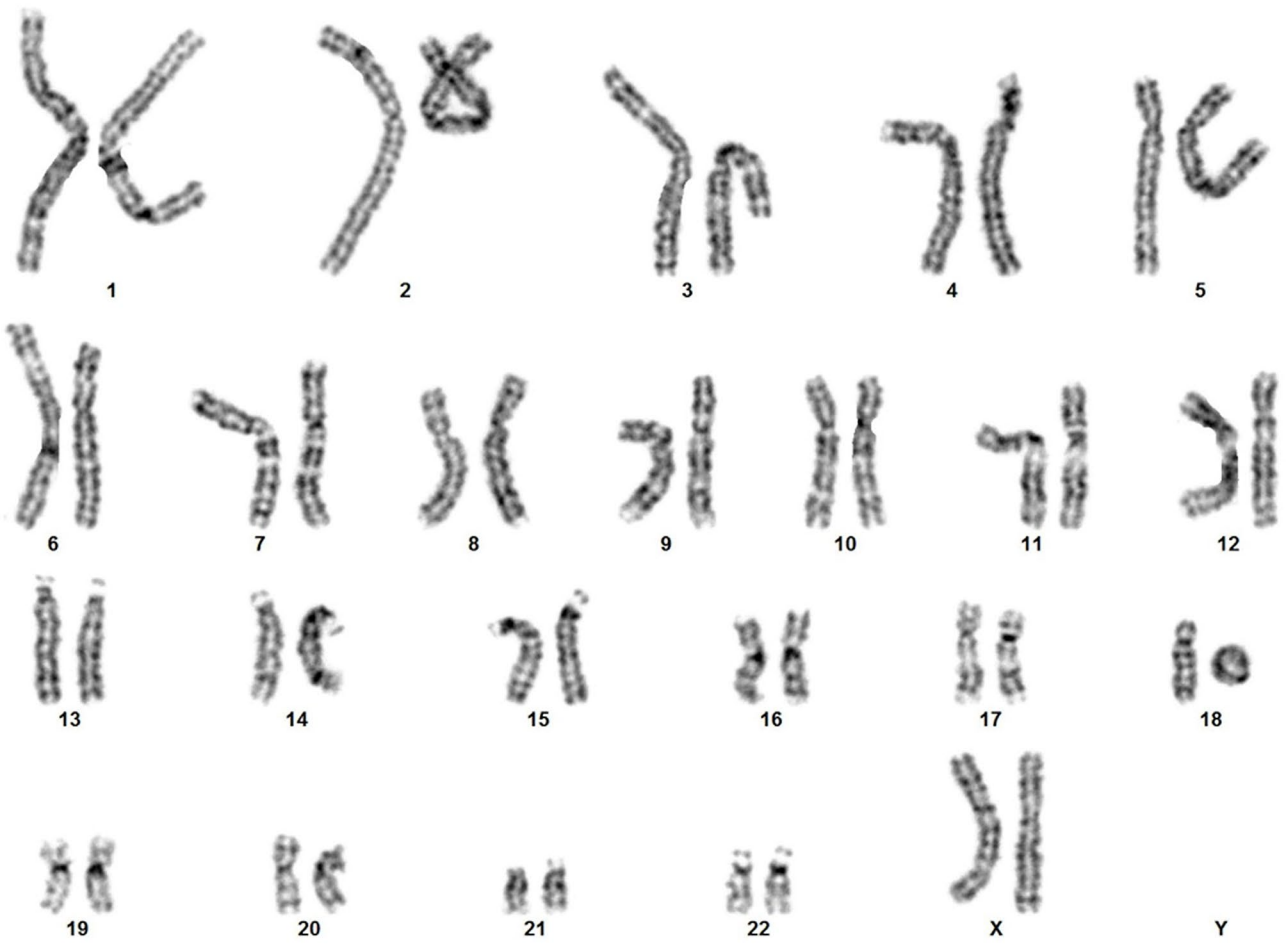
The karyotype illustrates the chromosomal makeup of our proband, showing the presence of a ring shaped chromosome 18

For the proband, standard chromosome analysis using GTG banding conducted on PHA-stimulated peripheral blood lymphocytes revealed a 46,XX, r(18)(p11.3q23) karyotype, according to ISCN 2020 (Fig. 1) in all the 20 metaphases analyzed, leading to the diagnosis of ring chromosome 18 syndrome.

### SNP-array

**Fig. 2:**
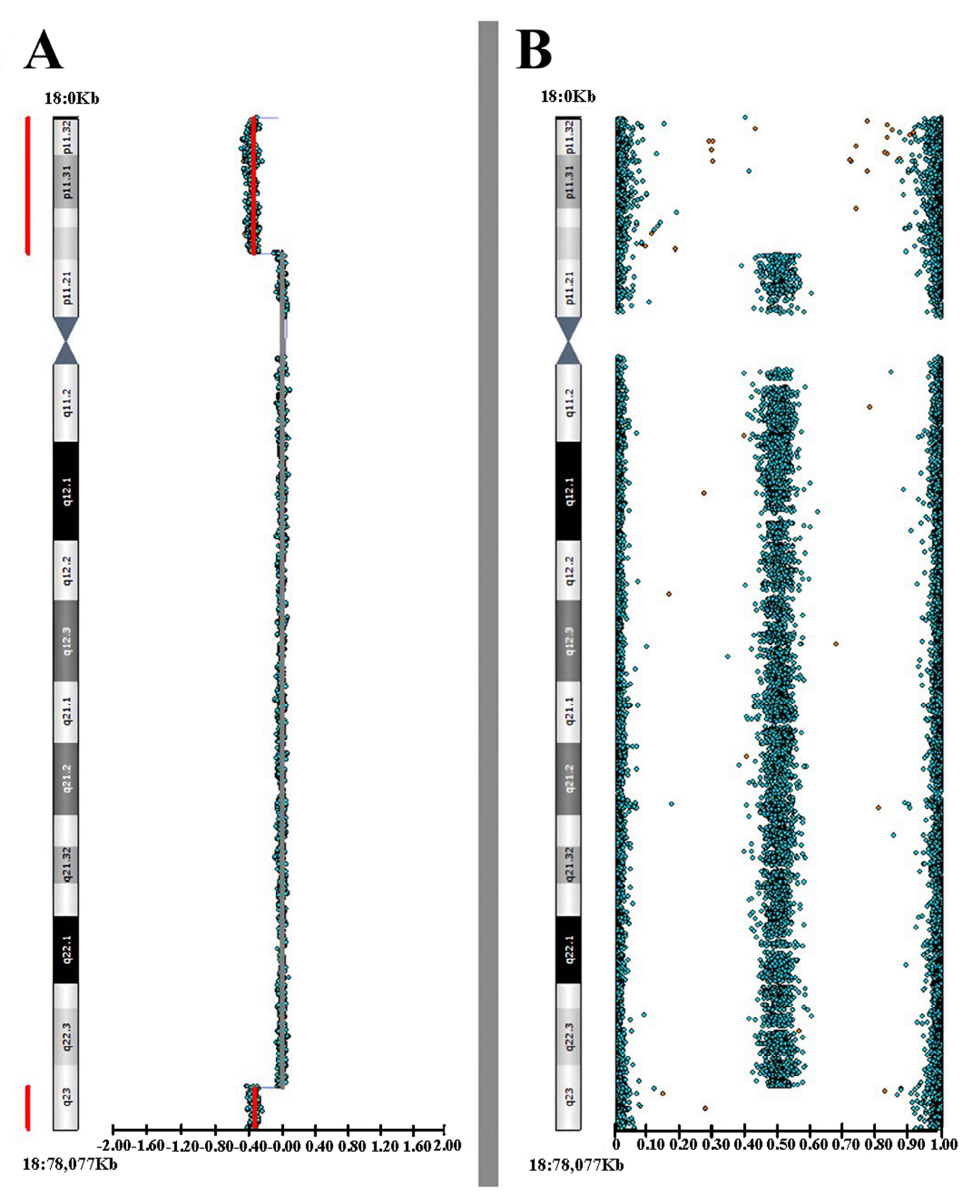
High-resolution SNP array analysis shows the presence of a deletion at 18p11.32p11.22 and a microdeletion at 18q23 in the proband. The left panel (**A**) showcases log R ratios (LRR) across chromosome 18, offering a normalized measure of signal intensity to evaluate CNVs. For each SNP, 0 indicates a typical, diploid copy number, while positive and negative values suggest copy number gains and losses, respectively. The right panel (**B**) depicts B allele frequency (BAF) values, assessing the proportion of two alleles present at a specific genomic locus. BAF values of 1, 0.5, and 0 correspond to homozygous for the reference allele (BB), heterozygous (AB), and homozygous for the alternative allele (AA) genotypes, respectively. The LogR plot displays a significant decrease in signal intensity in the regions 18p11.32p11.22 and 18q23, suggesting the presence of two deletions (copy number of x1). Likewise, the BAF plot confirms the loss of heterozygosity, affirming the existence of the two hemizygous deletions

SNP-array analysis revealed the presence of two *de novo* genomic imbalances: a deletion on the short arm of chromosome 18 (18p), specifically in the region 18p11.32p11.22 (arr[GRCh38] 18p11.32p11.22 (13034-10439156)x1 dn, according to ISCN 2020), spanning 10.4 Mb and including 36 OMIM genes, and a microdeletion of 3.2 Mb on the long arm of chromosome 18 (18q), in the region 18q23 (arr[GRCh38]18q23 (77042280–80257297)x1 dn, according to ISCN 2020), involving 10 OMIM genes (Fig. 2). Our results are consistent with the presence of a ring chromosome 18. The same test, performed on the parents, showed no aberrations in the regions examined, confirming that the rearrangements arose *de novo*.

### Genes analysis

The SNP array analysis enabled precise definition and characterization of the breakpoints (BP) of the r(18). Specifically, the BP on the short arm of chromosome 18 (18p) (chr18:10439156–10444658, GRCh38/hg38) does not encompass any genes, whereas the one on 18q (chr18:77035532–77042180, GRCh38/hg38) is located within intron 3 of the *MBP* gene.

The annotated pathogenicity of genes within the deleted regions was investigated using the Online Mendelian Inheritance in Man (OMIM) database. The analysis revealed that 22 out of the 36 OMIM genes within the 18p11.32p11.22 deletion are associated with 22 distinct phenotypes (Supplementary Table S1). Similarly, 3 of the 10 OMIM genes within the 18q23 deletion were found to be associated with 3 distinct phenotypes (Supplementary Table S1).

To gain a more comprehensive understanding of the repercussions of these genetic imbalances, a dosage sensitivity analysis was performed on the genes located within the rearranged regions of the patient’s genome using ClinGen. This analysis revealed that three genes (*LAMA1*,* LPIN2*,* NDUFV2*), included in the 18p11.32p11.22 deletion, exhibited an autosomal recessive haplotype (AR). In contrast, only one gene (*TGIF1*) within the same region demonstrated sufficient evidence for haploinsufficiency (HI) (Supplementary Table S2). Furthermore, in the 18q23 microdeletion, a single gene (*CTDP1*) was found to have an autosomal recessive haplotype (AR) (Supplementary Table S2).

## Discussion

In this study we present a case of a ring chromosome 18 identified in a pediatric patient via karyotyping, and high-resolution single nucleotide polymorphism (SNP) array analysis. The patient exhibited multiple autoimmune disorders, including hypothyroidism secondary to autoimmune thyroiditis and autoimmune hepatitis type II, in addition to a genetic predisposition for celiac disease and diabetes (IDDM). Comprehensive testing enabled early diagnosis and management of the patient’s complex medical conditions.

SNP array analysis identified two *de novo* genomic imbalances, a deletion in the region 18p11.32p11.22, and a microdeletion in the region 18q23, classified as pathogenic CNVs.

Notably, the BP on the short arm of chromosome 18 (18p) does not include any genes. This observation suggests that this BP does not directly disrupt any gene function, potentially mitigating its immediate effects on gene expression.

In contrast, the breakpoint on the long arm of chromosome 18 (18q) is located within intron 3 of the *MBP* (OMIM #159430) gene, which is not currently classified as morbid. However, the inclusion of *MBP* gene promoters in the deleted region suggests a potential impairment of their transcriptional activity. This could significantly affect the development and functionality of the proband’s nervous system, given the critical role of the MBP protein in the formation and stability of myelin [12]. The presence of hypotonia in our patient, along with MRI findings suggestive of gliosis, supports this hypothesis.

Integrated neuroimaging and molecular studies have revealed white matter abnormalities similar to those observed in our patient among individuals with 18q deletion syndrome, including five cases with ring chromosome 18 [5, 7, 9, 13, 14]. These abnormalities correlate with distal 18q deletions, particularly impacting 18q23 and resulting in hypomyelination due to hemizygosity of the *MBP* gene. In addition, Galanin (GAL), a neuropeptide composed of 29 amino acids, has been identified as a promoting factor in myelinogenesis within the nervous system [15]. The galanin receptor 1 gene (*GALR1;* OMIM #600377) is located immediately distal to the *MBP* gene on chromosome 18q [16]. Consequently, our patient also has a deletion of one copy of *GALR1*, which may further impair or alter myelination.

We performed a comparative analysis of our patient’s phenotype with those reported in the relevant literature, focusing on autoimmune manifestations and examining the potential role of genes on chromosome 18 in autoimmune processes (Supplementary Table S3). Our proband has several organ-specific autoimmune disorders including hypothyroidism secondary to autoimmune thyroiditis and autoimmune hepatitis type II, in addition to a genetic predisposition to celiac disease and type I diabetes mellitus (IDDM). Autoimmune thyroiditis is particularly prevalent among individuals with deletions or other structural abnormalities in chromosome 18, involving both 18p and 18q regions. This suggests that genes on both arms of chromosome 18 could play a role in maintaining immune tolerance​​ [9, 17, 18].

Hypothyroidism is a frequent complication in individuals with chromosome 18 anomalies, including r(18). The patients described in the studies by Ohkubo et al. (2012) and Lo-Castro et al. (2011) exhibited classic signs of autoimmune hypothyroidism, such as elevated thyroid-stimulating hormone (TSH) and the presence of thyroid antibodies (anti-TSH receptor, antithyroid peroxidase, and antithyroglobulin)​​. The underlying genetic deletions in r(18) patients are suspected to disrupt genes involved in thyroid regulation, predisposing them to autoimmune thyroiditis [18].

Liver dysfunction in the context of hypothyroidism is less commonly reported. However, it was observed in the patient described by Ohkubo et al. (2012). This patient presented with elevated liver enzymes (AST and ALT) which normalized upon achieving a euthyroid state with levothyroxine treatment. Although the exact mechanism is unclear, the normalization of liver enzymes with thyroid hormone replacement therapy suggests a possible link between low thyroid hormone levels and liver dysfunction. In the same study, it is speculated that this association might be mediated by the cellular stress caused by *NDUFV2* gene dysfunction. *NDUFV2*, located on chromosome 18p11.22, is a gene involved in mitochondrial complex I function. Mutations in this gene can lead to mitochondrial dysfunction, which can cause an imbalance between reactive oxygen species (ROS) and antioxidants, creating cellular stress that could potentially damage hepatocytes [18]. This process might also be responsible for the development of the autoimmune hepatitis type II observed in our patient.

Chromosome 18 abnormalities also correlate with an increased prevalence of IDDM [17], often co-occurring with autoimmune thyroiditis, particularly in individuals with deletions on chromosome 18q. These observations suggest a potential link between the IDDM6 susceptibility locus on chromosome 18q21 and the development of multiple autoimmune conditions [17]. The distinct spectrum of autoimmune disorders observed in individuals with chromosome 18 abnormalities suggests that specific genes or gene clusters on this chromosome play critical roles in immune regulation. For instance, the *CD226* Gly307Ser mutation on 18q22 has been implicated in predisposing individuals to type 1 diabetes, multiple sclerosis, and possibly autoimmune thyroid disease [19]. However, in our case the 18q21 and 18q22 regions encompassing the IDDM6 locus and the *CD226* gene are neither deleted nor mutated. This finding suggests that a distinct underlying mechanism may be responsible for the development of both autoimmune thyroiditis and IDDM in this patient.

Moreover, IgA deficiency, which is associated with increased autoimmunity, has been observed in patients with both 18p and 18q deletions and may contribute to the development of autoimmune disorders. However, this relationship is less clear in r(18) patients who do not exhibit IgA deficiency​ [17, 20].

## Conclusion

In conclusion, we report the diagnostic journey of a pediatric patient with ring chromosome 18 (r(18)) identified through karyotyping and further characterized by SNP array analysis. This comprehensive approach uncovered two *de novo* pathogenic CNVs – a deletion in 18p11.32p11.22 and a microdeletion in 18q23, highlighting the complexity of r(18) and its impact on immune regulation and nervous system development.

The patient’s clinical profile revealed significant autoimmune manifestations, including hypothyroidism due to autoimmune thyroiditis and autoimmune hepatitis type II, alongside genetic predispositions for celiac disease and insulin-dependent diabetes mellitus (IDDM).

Research into the genetic underpinnings of r(18) and its associated autoimmune disorders remains essential to pinpoint the specific genes involved and their functions in maintaining immune tolerance and preventing autoimmunity.

Moreover, this study calls for ongoing research to further elucidate the specific genetic pathways disrupted in r(18) and their contributions to the observed phenotypes. Understanding these mechanisms is crucial for developing targeted therapeutic strategies and improving outcomes for individuals with r(18).

## Methods

### Cytogenetic analysis and single nucleotide polymorphism (SNP) array

Conventional karyotype analysis was performed on GTG-banded chromosomes at a resolution of 400 bands, using phytohaemagglutinin (PHA)-stimulated peripheral blood lymphocytes.

Single Nucleotide Polymorphism (SNP) array analysis was conducted using the Illumina CytoSNP-850 K kit, which enabled the examination of approximately 850,000 SNPs. The SNPs were spaced with an average distance of 5 kb, and 1 kb in regions of genes considered significant by the International Collaboration for Clinical Genomics (ICCG) and the Cancer Cytogenomics Microarray Consortium (CCMC). This kit enabled the detection of copy number variants (CNVs), such as microdeletions and microduplications, involving a minimum of 10 consecutive SNP probes, achieving an average resolution of approximately 18 kb. The DNA used for this analysis was extracted from peripheral blood samples. The control sample used was CytoSNP-850Kv1-3_NextSeq_EMEAv1_ClusterFile. The BlueFuse Multi Software Edition 4.5, employing the BedArray v2 algorithm (hg19 release), was utilized for data analysis.

### Genes analysis

The pathogenicity of genes within the deleted regions was annotated using the Online Mendelian Inheritance in Man (OMIM) database. Additionally, a dosage sensitivity analysis was conducted on the genes located within the rearranged regions of the patient’s genome using the Clinical Genome Resource (ClinGen).

### Patient and public involvement statement

Patients or the public were not involved in the design, conduct, reporting, or dissemination plans of our research.

## Electronic supplementary material

Below is the link to the electronic supplementary material.

Supplementary Material 1

Supplementary Material 2

Supplementary Material 3

## Data Availability

The data supporting the findings of this study can be obtained from the corresponding authors, F.A. and A.C., upon reasonable request.
