# Supplementary material for: Clinical and genomic profiling of a patient with a de novo ring chromosome 18: a case report highlighting autoimmune and neurological implications
Source: Mol Cytogenet. 2024 Dec 5;17:31. doi: 10.1186/s13039-024-00700-5 (PMC11619688; doi:10.1186/s13039-024-00700-5)
Supplement: Supplementary file 1 — Supplementary Material 1 [file 13039_2024_700_MOESM1_ESM.pdf]

Table S1. Phenotype-associated genes contained in the regions 18p11.32p11.22 (chrchr18:13034-10439156, GRCh38/hg38) and 18q23 (chrchr18:77042280-80257297, GRCh38/hg38) deleted in the proband

Cytogenetic location	Genomic coordinates (From NCBI/GRCh38)	Gene/Locus	Gene/Locus name	Gene/Locus	Approved Symbol	Entrez Gene ID	Ensembl Gene ID	Comments	Phenotype	Phenotype MIM number	Inheritance
18p11.3-p11.2	chr18:1-15400000	AA1	Alopecia areata 1	104000		100034700		max lod at D18S967	Alopecia areata 1	104000	Mu
18p11.32-p11.31	chr18:1-7200000	DFNB46	Deafness, neurosensory, autosomal recessive 46	609647		449488		between D18S59 and D18S391	Deafness, autosomal recessive 46	609647	AR
18p11	chr18:1-18500000	DYT15	Dystonia-15, myoclonic	607488		317714			Dystonia-15, myoclonic	607488	AD
18p	chr18:1-18500001	DYT7	Dystonia-7 (torsion dystonia, adult-onset, focal)	602124		1866			Dystonia-7, torsion	602124	AD
18pter-p11.21	chr18:1-15400000	ERV1	Oncogene ERV1; endogenous retrovirus-1	131150							
18p11	chr18:1-18500000	IBD21	Inflammatory bowel disease 21	612354		100192312		associated with rs2542151	{Inflammatory bowel disease 21}	612354	AD
18p	chr18:1-18500001	MAFD1, BPAD, MD1	Major affective disorder 1	125480		4095		?also 18q	{Major affective disorder 1}	125480	AD
18p11.3	chr18:1-7200000	MRT19	Intellectual developmental disorder, autosomal recessive 19	614343		100852395		between rs4606805 and rs1787846	Intellectual developmental disorder, autosomal recessive 19	614343	AR
18p11.3	chr18:1-7200000	POROK6, DSAP4	Porokeratosis 6	612353		100196911		between telomere and D18S391	Porokeratosis 6, multiple types	612353	AD
18p	chr18:1-18500001	SCZD8	Schizophrenia susceptibility locus, chromosome 18-related	603206		8806			{Schizophrenia}	181500	AD
18p11.32	chr18:158557-214629	USP14, TGT	Ubiquitin-specific protease 14	607274	USP14	9097	ENSG00000101557,ENST00000261601;;;ENST00000261601				
18p11.32	chr18:214520-268047	THOC1, HPR1, DFNA86	THO complex 1 (nuclear matrix protein p84)	606930	THOC1	9984	ENSG00000079134,ENST00000261600;;;ENST00000261600	mutation identified in 1 DFNA86 family	?Deafness, autosomal dominant 86	620280	AD
18p11.32	chr18:316737-500701	COLEC12, SRCL, CLP1	Collectin 12	607621	COLEC12	81035	ENSG00000158270,ENST00000400256;;;ENST00000400256				
18p11.32	chr18:580380-582114	CETN1, CEN1	Centrin-1	603187	CETN1	1068	ENSG00000177143,ENST00000327228;;;ENST00000327228				
18p11.32	chr18:596988-650182	CLUL1	Clusterin-like protein 1	616990	CLUL1	27098	ENSG00000079101,ENST00000692774;;;ENST00000692774				
18p11.32	chr18:657653-673578	TYMS, TS, TMS, DKCD	Thymidylate synthase	188350	TYMS	7298	ENSG00000176890,ENST00000323274;;;ENST00000323274	DKCD is digenic with C-A-ins haplotype	Dyskeratosis congenita, digenic	620040	DD
18p11.32	chr18:662986-712630	ENOSF1, RTS	Enolase superfamily member 1	607427	ENOSF1	55556	ENSG00000132199,ENST00000647584;;;ENST00000647584				
18p11.32	chr18:721588-812753	YES1	Oncogene YES-1	164880	YES1	7525	ENSG00000176105,ENST00000314574;;;ENST00000314574	<50 kb from TYMS			
18p11.32	chr18:904411-912172	ADCYAP1	Adenylate cyclase activating polypeptide-1 (pituitary)	102980	ADCYAP1	116	ENSG00000141433,ENST00000450565;;;ENST00000450565				
18p11.32	chr18:2537530-2571505	METTL4	Methyltransferase 4, N6-adenosine	619626	METTL4	64863	ENSG00000101574,ENST00000574538;;;ENST00000574538				
18p11.32	chr18:2571557-2616635	NDC80, KNTC2, HEC	NDC80 kinetochore complex component	607272	NDC80	10403	ENSG00000080986,ENST00000261597;;;ENST00000261597				
18p11.32	chr18:2655726-2805017	SMCHD1, KIAA0650, BAMS	Structural maintenance of chromosomes flexible hinge domain-containing protein 1	614982	SMCHD1	23347	ENSG00000101596,ENST00000320876;;;ENST00000320876		Bosma arhinia microphthalmia syndrome	603457	AD
18p11.32	chr18:2655726-2805017	SMCHD1, KIAA0650, BAMS	Structural maintenance of chromosomes flexible hinge domain-containing protein 1	614982	SMCHD1	23347	ENSG00000101596,ENST00000320876;;;ENST00000320876		Facioscapulohumeral muscular dystrophy 2, digenic	158901	DD
18p11.32-p11.31	chr18:2846232-2916003	EMILIN2	Elastin macrofibril interfacer 2	608928	EMILIN2	84034	ENSG00000132205,ENST00000254528;;;ENST00000254528				
18p11.31	chr18:2900001-7200000	MYP2	Myopia, high grade, autosomal dominant 1	160700		4658			Myopia 2	160700	AD
18p11.31	chr18:2916994-3013144	LPIN2, MJDS, CRMO1	Lipin 2	605519	LPIN2	9663	ENSG00000101577,ENST00000677752;;;ENST00000677752		Majeed syndrome	609628	
18p11.31	chr18:3066807-3247376	MYOM1, SKELEMIN	Myomesin 1	603508	MYOM1	8736	ENSG00000101605,ENST00000356443;;;ENST00000356443				
18p11.31	chr18:3262133-3278461	MYL12B, MRLC2	Myosin, light chain 12B, regulatory	609211	MYL12B	103910	ENSG00000118680,ENST00000237500;;;ENST00000237500				
18p11.31	chr18:3412009-3459978	TGIF1, HPE4	TG-interacting factor 1	602630	TGIF1	7050	ENSG00000177426,ENST00000343820;;;ENST00000343820		Holoprosencephaly 4	142946	AD
18p11.31	chr18:3496032-4455307	DLGAP1, DAP1, DLGAP1A, DLGAP1B	Discs large-associated protein 1	605445	DLGAP1	9229	ENSG00000170579,ENST00000315677;;;ENST00000315677				
18p11.31	chr18:5142911-5197691	C18orf42	Chromosome 18 open reading frame 42	616427	AKAIN1	642597	ENSG00000231824,ENST00000434239;;;ENST00000434239				
18p11.31	chr18:5289022-5297053	ZFP161, ZF5	Zinc finger protein-161	602126	ZBTB14	7541	ENSG00000198081,ENST00000651870;;;ENST00000651870				
18p11.31	chr18:5392386-5630663	EPB41L3, DAL1	Erythrocyte membrane protein band 4.1-like 3	605331	EPB41L3	23136	ENSG00000082397,ENST00000341928;;;ENST00000341928				
18p11.31	chr18:5954717-6415259	L3MBTL4	L3MBTL histone methyl-lysine-binding protein 4	617135	L3MBTL4	91133	ENSG00000154655,ENST00000317931;;;ENST00000317931				
18p11.31	chr18:6729716-6915716	ARHGAP28, KIAA1314	RHO GTPase-activating protein 28	610592	ARHGAP28	79822	ENSG00000088756,ENST00000383472;;;ENST00000383472				
18p11.31	chr18:6941742-7117797	LAMA1, PTBHS	Laminin, alpha-1	150320	LAMA1	284217	ENSG00000101680,ENST00000389658;;;ENST00000389658		Poretti-Boltshauser syndrome	615960	AR
18p11.23-q12.2	chr18:7200001-39500000	ANIC	Anosmia, isolated congenital	107200		550625		max lod at D18S1108	Anosmia, isolated congenital	107200	AD
18p11.2	chr18:7200001-15400000	DYX6, DYXQTL18	Dyslexia, susceptibility to, 6	606616		266691			{Dyslexia, susceptibility to, 6}	606616	
18p11.2	chr18:7200001-15400000	FEB6	Febrile seizures, familial, 6	609253		619397		max lod at D18S1158	Febrile seizures, familial, 6	609253	AD
18p11.23	chr18:7200001-8500000	PSORS10	Psoriasis susceptibility 10	612410		503613		between D18S63 and D18S967	{Psoriasis susceptibility 10}	612410	
18p11.23	chr18:7567316-8406856	PTPRM, PTPRL1, RPTPM	Protein tyrosine phosphatase, receptor type, mu polypeptide	176888	PTPRM	5797	ENSG00000173482,ENST00000580170;;;ENST00000580170				
18p11.22	chr18:8609437-8639383	RAB12	Ras-associated protein RAB12	616448	RAB12	201475	ENSG00000206418,ENST00000649141;;;ENST00000649141				
18p11.22	chr18:8695856-8707621	GACAT2, MTCL1AS1	Gastric cancer-associated transcript 2, noncoding	616131	GACAT2	100287082	ENSG00000265962,ENST00000579368				
18p11.22	chr18:8705556-8832778	MTCL1, KIAA0802	Microtubule crosslinking factor 1	615766	MTCL1	23255	ENSG00000168502,ENST00000695636;;;ENST00000695636				
18p11.22	chr18:9102699-9134341	NDUFV2, MC1DN7	NADH-ubiquinone oxidoreductase core subunit V2	600532	NDUFV2	4729	ENSG00000178127,ENST00000318388;;;ENST00000318388	pseudogene on 19q13.3-qter	Mitochondrial complex I deficiency, nuclear type 7	618229	AR
18p11.22	chr18:9136781-9285985	ANKRD12, ANCO1, KIAA0874	Ankyrin repeat domain-containing protein 12	610616	ANKRD12	23253	ENSG00000101745,ENST00000262126;;;ENST00000262126				
18p11.22	chr18:9334773-9402420	TWSG1, TSG	Twisted gastrulation BMP modulator 1	605049	TWSG1	57045	ENSG00000128791,ENST00000262120;;;ENST00000262120				
18p11.22	chr18:9475009-9538114	RALBP1, RLIP76	RALA-binding protein 1	605801	RALBP1	10928	ENSG00000017797,ENST00000383432;;;ENST00000383432				
18p11.22	chr18:9546794-9617199	PPP4R1, PP4R1	Protein phosphatase 4, regulatory subunit 1	604908	PPP4R1	9989	ENSG00000154845,ENST00000400556;;;ENST00000400556				
18p11.22	chr18:9708301-9862551	RAB31	Ras-associated protein RAB31	605694	RAB31	11031	ENSG00000168461,ENST00000578921;;;ENST00000578921				
18p11.22	chr18:9885973-9889275	TXNDC2, SPTRX1	Thioredoxin domain-containing protein 2	617790	TXNDC2	84203	ENSG00000168454,ENST00000357775;;;ENST00000357775				
18p11.22	chr18:9914016-9960021	VAPA, VAP33	VAMP-associated protein A	605703	VAPA	9218	ENSG00000101558,ENST00000400000;;;ENST00000400000				
18q	chr18:18500001-80373285	OHDS	Orthostatic hypotensive disorder of Streeten	143850		50948			Orthostatic hypotensive disorder of Streeten	143850	AD
18q23	chr18:76978833-77133708	MBP	Myelin basic protein	159430	MBP	4155	ENSG00000197971,ENST00000355994;;;ENST00000355994				
18q23	chr18:77249848-77277900	GALR1, GALNR1, GALNR	Galanin receptor 1	600377	GALR1	2587	ENSG00000166573,ENST00000299727;;;ENST00000299727				
18q23	chr18:78979818-78998969	SALL3	Sal-like 3	605079	SALL3	27164	ENSG00000256463,ENST00000537592;;;ENST00000537592				
18q23	chr18:79069394-79378283	ATP9B	ATPase, class II, type 9B	614446	ATP9B	374868	ENSG00000166377,ENST00000426216;;;ENST00000426216				
18q23	chr18:79395930-79529323	NFATC1	Nuclear factor of activated T cells, cytoplasmic, calcineurin-dependent 1	600489	NFATC1	4772	ENSG00000131196,ENST00000427363;;;ENST00000427363				
18q23	chr18:79676768-79756625	CTDP1, FCP1, CCFDN	C-terminal domain of RNA polymerase II subunit A, phosphatase of, subunit 1	604927	CTDP1	9150	ENSG00000060069,ENST00000613122;;;ENST00000613122		Congenital cataracts, facial dysmorphism, and neuropathy	604168	AR
18q23	chr18:79797938-79900100	KCNG2	Potassium channel, voltage-gated, subfamily G, member 2	605696	KCNG2	26251	ENSG00000178342,ENST00000316249;;;ENST00000316249				
18q23	chr18:79970813-80033936	TXNL4A, DIM1, BMKS	Thioredoxin-like 4A	611595	TXNL4A	10907	ENSG00000141759,ENST00000269601;;;ENST00000269601		Burn-McKeown syndrome	608572	AR
18q23	chr18:80034434-80050651	RBFA	Ribosome-binding factor A	620768	RBFA	79863	ENSG00000101546,ENST00000306735;;;ENST00000306735				
18q23	chr18:80109262-80140346	ADNP2, KIAA0863	Activity-dependent neuroprotector homeobox 2	617422	ADNP2	22850	ENSG00000101544,ENST00000262198;;;ENST00000262198				
18q23	chr18:80157232-80247514	PARD6G	partitioning-defective protein 6, C. elegans, homolog of	608976	PARD6G	84552	ENSG00000178184,ENST00000353265;;;ENST00000353265				

Inheritance Abbreviations

?AD	?Autosomal dominant
?XLR	?X-linked recessive
AD	Autosomal dominant
AR	Autosomal recessive
PD	Pseudoautosomal dominant
PR	Pseudoautosomal recessive
DD	Digenic dominant
DR	Digenic recessive
ICB	Inherited chromosomal imbalance
IC	Isolated cases
Mi	Mitochondrial
Mu	Multifactorial
SMo	Somatic mosaicism
SMu	Somatic mutation
XL	X-linked
XLD	X-linked dominant
XLR	X-linked recessive
YL	Y-linked
