# Supplementary material for: Clinical and genomic profiling of a patient with a de novo ring chromosome 18: a case report highlighting autoimmune and neurological implications
Source: Mol Cytogenet. 2024 Dec 5;17:31. doi: 10.1186/s13039-024-00700-5 (PMC11619688; doi:10.1186/s13039-024-00700-5)
Supplement: Supplementary file 2 — Supplementary Material 2 [file 13039_2024_700_MOESM2_ESM.pdf]

**Table S2. Dosage sensitive genes embedded in the deleted regions of our patient.**

| | #chrom | chromStart | chromEnd | name/geneSymbol | haploScore | haploDescription | url | geneId | cytoBand |
|---|---|---|---|---|---|---|---|---|---|
| DELETION 18p11.32p11.22 | chr18 | 3412009 | 3459978 | TGFB induced factor homeobox 1/TGIF1 | 3 | sufficient evidence for Haploinsufficiency | https://search.clinicalgenome.org/kb/gene-dosage/TGIF1 | 7050 | 18p11.31 |
| | chr18 | 2916994 | 3013144 | lipin 2/LPIN2 | 30 | Gene Associated with Autosomal Recessive Phenotype | https://search.clinicalgenome.org/kb/gene-dosage/LPIN2 | 9663 | 18p11.31 |
| | chr18 | 6941742 | 7117797 | laminin subunit alpha 1/LAMA1 | 30 | Gene Associated with Autosomal Recessive Phenotype | https://search.clinicalgenome.org/kb/gene-dosage/LAMA1 | 284217 | 18p11.31 |
| | chr18 | 9102699 | 9134341 | NADH:ubiquinone oxidoreductase core subunit V2/NDUFV2 | 30 | Gene Associated with Autosomal Recessive Phenotype | https://search.clinicalgenome.org/kb/gene-dosage/NDUFV2 | 4729 | 18p11.22 |
| DELETION 18q23 | #chrom | chromStart | chromEnd | name/geneSymbol | haploScore | haploDescription | url | geneId | cytoBand |
| | chr18 | 77249848 | 77277900 | galanin receptor 1/GALR1 | 0 | No Evidence for Haploinsufficiency | https://search.clinicalgenome.org/kb/gene-dosage/GALR1 | 2587 | 18q23 |
| | chr18 | 79676768 | 79756625 | CTD phosphatase subunit 1/CTDP1 | 30 | Gene Associated with Autosomal Recessive Phenotype | https://search.clinicalgenome.org/kb/gene-dosage/CTDP1 | 9150 | 18q23 |

**Dosage Scores** are used to classify the evidence of the supporting dosage sensitivity map:
**0** - no evidence available
**1** - little evidence for dosage pathogenicity
**2** - some evidence for dosage pathogenicity
**3** - sufficient evidence for dosage pathogenicity
**30** - gene associated with autosomal recessive phenotype
**40** - dosage sensitivity unlikely
