# Supplementary material for: Clinical and genomic profiling of a patient with a de novo ring chromosome 18: a case report highlighting autoimmune and neurological implications
Source: Mol Cytogenet. 2024 Dec 5;17:31. doi: 10.1186/s13039-024-00700-5 (PMC11619688; doi:10.1186/s13039-024-00700-5)
Supplement: Supplementary file 3 — Supplementary Material 3 [file 13039_2024_700_MOESM3_ESM.pdf]

Table S3. Clinical features of the proband and previously reported ring 18 cases

								Neurologic Disorders					
**Patient**	**GTG banding**	**SNP-array /arraCGH GRCh38/hg18**	**Ring origin**	**Age**	**Developmental delay, Intellectual disabilities and Neurodevelopmental disorders**	**Microcephaly**	**Cerebral MRI**	**Seizures**	**Hypotonia**	**Endocrine and Immunological disease**	**Spleen and Liver**	**Reference**	
1 (present study)	46,XX,r(18)(p11.3q23)	**SNP-array**<br>18p11.32p11.22(13034-10439156)x1 dn<br>18q23(77042280-80257297)x1 dn	*De novo*	2 yo	+	+	Slight T2 hyperintensity in periventricular and subcortical parietal frontal bilateral white matter<br>(possible ischemic outcomes);<br><br>On March 14th 2024: "presence of some millimetric hyperdense areas in FLAIR/T2, iso-hypointense in T1 and without impregnation after intravenous contrast medium. in the deep white matter of the corona radiates and semioval centers, of gliotic reparative significance" → gliosis	N/A	+	IDDM<br>Autoimmune Hepatopathy (type II)<br>Hypothyroidism and autoimmune thyroiditis<br>Increased IgG level<br>Celiac predisposition	Autoimmune Hepatopathy (type II)	present study	Case 1
2	46,XX, r(18) (p11.32q23)<br>45,XX,-18?	**SNP-array**<br>18p11.32-p11.21 deletion (chr18: 12842-11176069)<br>18q21.32-q23 deletion (chr18:60995190-80256699)<br>BP: chr18: 11172225 and 60941807	*De novo*	Termination of the pregnancy at 22 weeks' gestation	N/A	N/A	N/A	N/A	N/A	N/A	N/A	Ji et al, 2015<br>DOI 10.1186/s12881-015-0206-x	Case 1
3	46, XX, r(18)? (p11.32q23)	**SNP-array**<br>18p11.32 deletion (chr18: 12842-2548129)<br>18q22.1-q23 deletion (chr18:65462437-80256699)<br>BP:chr18: 2551698-65448248	*De novo*	8-month-old	+	+	No structural abnormalities	N/A	N/A	N/A	N/A		Case 2
4	46,XY,r(18) (p11.1q21.31)	**SNP-array**<br>18p11.1(10001-15410817)x1<br>18q21.31(57708421-80257297)x1	Maternal	9 yo	+	+	N/A	-	+	IgA deficiency<br>Growth hormone deficiency	N/A	Spreiz et al, 2013<br>http://dx.doi.org/10.1016/j.jpeds.2013.06.005	Case 1
5	46,XX,r(18) (p11.32q22.1)	**SNP-array**<br>18p11.32(12842-1601783)x1<br>18q22.1(65246213-80257297)x1	Maternal	22 yo	+	-	Normal	Neonatal	Neonatal	N/A	N/A		Case 2
6	46,XX,r(18) (p11.1q23)	**SNP-array**<br>18p11.1(10001-15410817)x1<br>18q23(77009001-80257297)x1	Paternal	7 yo	+	+	N/A	+	-	N/A	N/A		Case 3
7	46,XX,r(18) (p11.31q21.2)	**SNP-array**<br>18p11.32p11.31(12842-6600606)x1<br>18p11.31p11.23(54272075-78356152)x3<br>18q21.2q23(6603955-7962086)x1<br>Xp22.33(439373-900821)x1	Paternal	14 yo	+	+	N/A	N/A	+	N/A	N/A		Case 4
8	46,XX,r(18) (p11.32q21.2)	**SNP-array**<br>18p11.32p11.32(12842-2452253)x1<br>18p11.32p11.31(51343832-80257297) x3<br>18q21.2q23(2454531-4517485)x1	Paternal	1 yo	+	+	Normal	+	N/A	N/A	N/A		Case 5
9	46,XX,r(18) (p11.32q22.1)	**SNP-array**<br>18p11.32p11.31(67386560-80257297)x3<br>18q22.1q23(12842-5832746)x1	Paternal	39 yo	+	-	N/A	-	+	N/A	N/A		Case 6
10	46,XY,r(18) (p11.32q22.2)	**SNP-array**<br>18p11.32(11542-1383477)x1<br>18q22.2q23(69199648-80257174)x1	N/A	11 yo	+	+	Reduced white substance	-	+	IgA deficiency<br>Subclinical hypothyroidism	N/A		Case 7
11	46,XX,r(18) (18p11.32q21.33)	**SNP-array**<br>18q21.33q23(61641439-80257174)x1	N/A	7 yo	+	+	Ventricular asymmetry	-	+	IgA deficiency	N/A		Case 8
12	46,XX,r(18) (p11.32q23)	**SNP-array**<br>18p11.32p11.21(11542-15249168)x1<br>18q23(78570387-80257174)x1	N/A	13 yo	+	-	Hypomyelination/demyelination or gliosis	Before 1.8 months	Infancy	IgA deficiency (bronchitis)	Chronic hepatitis (since baby)<br>Hepatosplenomegaly		Case 9
13	46, XX, r(18) (p11.32; q22.2)	N/A	*De novo*	27 yo	+	+	N/A	N/A	+	Rheumatoid Arthritis<br>Systemic Lupus Erythematosus<br>Crohn's disease	N/A	Rezaeizadeh et al, 2022<br>https://doi.org/10.1007/s11033-021-06933-6	
14	46,XY,r(18)(p11;q21)<br>46,XY	Results not found<br><br>*The array results in the patient's family demonstrate a postzygotic formation of the r(18) in the mother, and in the son a maternal gamete carrying the r(18) and the normal chromosome 18 from which the r(18) originated, and a subsequent loss of either the r(18) or the maternal chromosome 18, respectively, as the most likely mechanism of formation	Most likely maternal origin	9 months to 23 yo	+	+	(CT) frontotemporal atrophy and a large cisterna magna	N/A	+	N/A	N/A	Balci et al, 2014<br>DOI 10.1002/ajmg.a.36496<br>Balci et al, 2010<br>DOI: 10.1002/ajmg.a.33868	Case 1
15	46,XX,r(18)(p11.32q22.2)<br>45,XX,-18<br>and trisomy for double ring chromosomes (<1%)	N/A	N/A	N/A	+	N/A	Hypomyelination/demyelination or gliosis	N/A	Since birth	N/A	N/A	Lammert et al, 2019<br>DOI: 10.1002/ccr3.2426	
16	45,XX,-18<br>46,XX,r(18)(p11.3q23)<br>46,XX	**Array-CGH**<br>18p11.32p11.31(180299-4453435)x1<br>18q22.1q23(66190016-80224243)x1	*De novo*	9 yo	+	mild	Hypomyelination/demyelination or gliosis	Focal seizures	N/A	Hypothyroidism<br>Celiac disease	Mild hepatomegaly<br>Splenomegaly	Lo-Castro et al, 2011<br>10.1016/j.ejmg.2011.02.004	Case 1
17	46, XY, r(18)(p11.2q23)	N/A	*De novo*	2 yo	+	N/A	Slightly enlarged anterior pituitary	N/A	+	High IgG<br>IgA deficiency<br>Autoimmune hypothyroidism	Liver dysfunction	Ohkubo et al, 2012<br>DOI: 10.1089/thy.2011.0521	
18	46,XX,r(18)	N/A	*De novo*	12 yo	+	N/A	Mild cerebral atrophy	At 11 yo	N/A	Subclinical hypothyroidism	N/A	Wang et al, 2021<br>https://doi.org/10.1007/s10072-021-05143-z	
19	46,XY,r(18)(p11.21q21.2)<br>45,XY,-18<br>46,XY<br>46,XY,idic r(18)(p11.21q21.2;p11.21q21.2)	**Array-CGH**<br>18p11.32p11.21 (10001-14951331) × 1<br>18q21.2q23 (50753062-80258298) × 1	*De novo*	Termination of the pregnancy at 38 weeks' gestation	N/A	+	Ventriculomegaly	N/A	+	N/A	N/A	Chen et al, 2010<br>doi: 10.1016/S1028-4559(10)60069-1	Case 1
20	46, XY, r(18) (p11; q22)	**Array-CGH**coordinates not reported<br>14.9 Mb deletion at 18p11.32–p11.21<br>14.4 Mb deletion at 18q22.1–q23	*De novo*	14 yo	+	N/A	Ventriculomegaly and diffuse hyperintensity in the white matter	N/A	+	N/A	N/A	Anzai et al, 2016<br>doi: 10.1111/ped.13043	Case 1
21	46,XY,r(18) (p11.32q22.3)	**Array-CGH**<br>18p11.32(142096-447282)<br>18q22.3q23(72715405-80224243)	*De novo*	_	+	+	N/A	N/A	N/A	N/A	N/A	Zlotina et al,2016<br>DOI 10.1186/s13039-016-0229-9	Case 1
22	46, XY, r(18) (p11.32 q21.32)	N/A	*De novo*	2,5 yo	+	N/A	N/A	N/A	N/A	N/A	N/A	Heydari et al, 2014<br>PMID: 25635256	Case 1
23	46,XY,r(18)	N/A	*De novo*	_	-	N/A	N/A	N/A	N/A	N/A	N/A	Dobos et al, 2004<br>https://doi.org/10.1080/09723757.2004.11885892	Case 1
24	46,XX,r(18)		*De novo*	_	N/A	+	N/A	N/A	+	N/A	N/A		Case 2
25	46,XX,r(18)		*De novo*	_	+	+	N/A	N/A	+	N/A	N/A		Case 3
26	46,XX,r(18)<br>46,XX,der(18)		*De novo*	_	+	N/A	N/A	N/A	+	N/A	N/A		Case 4
